# LAMTOR2 (p14) Controls B Cell Differentiation by Orchestrating Endosomal BCR Trafficking

**DOI:** 10.3389/fimmu.2019.00497

**Published:** 2019-03-18

**Authors:** Marcin Łyszkiewicz, Daniel Kotlarz, Natalia Ziȩtara, Gudrun Brandes, Jana Diestelhorst, Silke Glage, Elias Hobeika, Michael Reth, Lukas A. Huber, Andreas Krueger, Christoph Klein

**Affiliations:** ^1^Institute of Immunology, Hannover Medical School, Hannover, Germany; ^2^Department of Pediatrics, Dr. von Hauner Children's Hospital, University Hospital, Ludwig-Maximilians-University Munich, Munich, Germany; ^3^Institute of Neuroanatomy and Cell Biology, Hannover Medical School, Hannover, Germany; ^4^Institute of Laboratory Animal Science, Hannover Medical School, Hannover, Germany; ^5^Institute of Immunology, Ulm University, Ulm, Germany; ^6^Max Planck Institute of Immunobiology and Epigenetics, Freiburg, Germany; ^7^Division of Cell Biology, Biocenter, Medical University of Innsbruck, Innsbruck, Austria; ^8^Institute for Molecular Medicine, Goethe-University Frankfurt, Frankfurt am Main, Germany

**Keywords:** B cells, B-cell antigen receptor, LAMTOR2, signal transduction, trafficking, p14

## Abstract

B-cell development and function depend on stage-specific signaling through the B-cell antigen receptor (BCR). Signaling and intracellular trafficking of the BCR are connected, but the molecular mechanisms of this link are incompletely understood. Here, we investigated the role of the endosomal adaptor protein and member of the LAMTOR/Ragulator complex LAMTOR2 (p14) in B-cell development. Efficient conditional deletion of LAMTOR2 at the pre-B1 stage using *mb1*-Cre mice resulted in complete developmental arrest. Deletion of LAMTOR2 using *Cd19*-Cre mice permitted analysis of residual B cells at later developmental stages, revealing that LAMTOR2 was critical for the generation and activation of mature B lymphocytes. Loss of LAMTOR2 resulted in aberrant BCR signaling due to delayed receptor internalization and endosomal trafficking. In conclusion, we identify LAMTOR2 as critical regulator of BCR trafficking and signaling that is essential for early B-cell development in mice.

## Introduction

Signals from the B cell antigen receptor (BCR) determine development and function of B cells. During development, a pre-BCR is first assembled by pairing immunoglobulin (Ig) heavy chains with surrogate light chains (VpreB and λ5). Thus, signaling through the pre-BCR indicates successful Ig heavy chain gene rearrangement and promotes transition from the pro-B/pre-B1 to the pre-B2 developmental stage (1–3). Phenotypically, these stages can be discriminated by expression of CD117 and CD25 on the surface of pro-B/pre-B1 and pre-B2 cells, respectively (4). Later during development BCR signaling serves to monitor cells for productive Ig light chain rearrangements and to remove autoreactive B cells in bone marrow and spleen (4). Finally, recognition of antigen by the BCR in combination with T cell help results in B cell activation and eventually differentiation into plasma cells, production of antibodies and memory formation. Signal transduction through the BCR comprises proximal events of tyrosine phosphorylation resulting in downstream activation of multiple signaling cascades including PLCγ/Ca^2+^/NF-κB/NFAT, Rho family, PI3-K/Akt as well as the Ras/Raf-1/Erk pathway (5). Receptor internalization constitutes another consequence of BCR engagement (6). However, the interdependence between signaling and internalization of the receptor remains to be fully characterized. On the one hand, it has been reported that both processes are mutually exclusive (7). On the other hand, it has been proposed that internalization of the BCR via the endosomal route is required for proper spatial organization of signal transduction (8). Thus, inhibition of BCR endocytosis resulted in dysregulation of kinase activation.

LAMTOR2 (p14) has been described as an endosomal adaptor protein and forms a part of the late endosomal/lysosomal adaptor and MAPK and mTOR activator (LAMTOR)/Ragulator complex at late endosomes (9). LAMTOR2 contributes to spatial organization of the endosomal compartment and regulates proliferation. As a consequence, constitutive deletion of LAMTOR2 in mice results in embryonic lethality (10). Together with its partner MP-1, LAMTOR2 provides a scaffold for recruitment of the MAP kinase Erk to endosomes (11). Spatial compartmentalization of Erk has been suggested to provide context-dependent specificity for this signaling pathway (12). Deletion of LAMTOR2 results in complete abrogation of the LAMTOR/Ragulator complex (10, 13). The function of the LAMTOR/Ragulator complex in B cells has not been explored.

Of note, humans carrying a homozygous mutation in the 3′ untranslated region of the gene encoding LAMTOR2, resulting in massively reduced expression of protein, display partial albinism, short stature as well as complex hematologic, and immunologic defects, including congenital neutropenia, defects in cytotoxic T lymphocytes, and defects in B-cell development and function (14).

Given the emerging role of spatial compartmentalization of signaling downstream of the BCR and the yet mechanistically uncharacterized B-lineage defect in humans carrying a mutation in LAMTOR2, we hypothesized that the LAMTOR/Ragulator complex might affect B-cell development and function. To test this hypothesis, we employed B-lineage specific deletion of the gene encoding LAMTOR2 gene in mice to study the function of the LAMTOR/Ragulator complex in B cells. Loss of LAMTOR2 in the B lineage resulted in a profound block at the pre-BI to pre-B2 developmental transition due to a defect in pre-BCR signaling. Furthermore, LAMTOR2-deficient B cells displayed aberrant kinase activation as well as dysregulated trafficking of the BCR upon stimulation. We conclude that aberrant BCR signaling due to a defect in BCR internalization in the absence of LAMTOR2 provides an explanation for the developmental defect observed in the B lineage.

## Results

### LAMTOR2 Is Critical for B-cell Development

**Figure 1:**
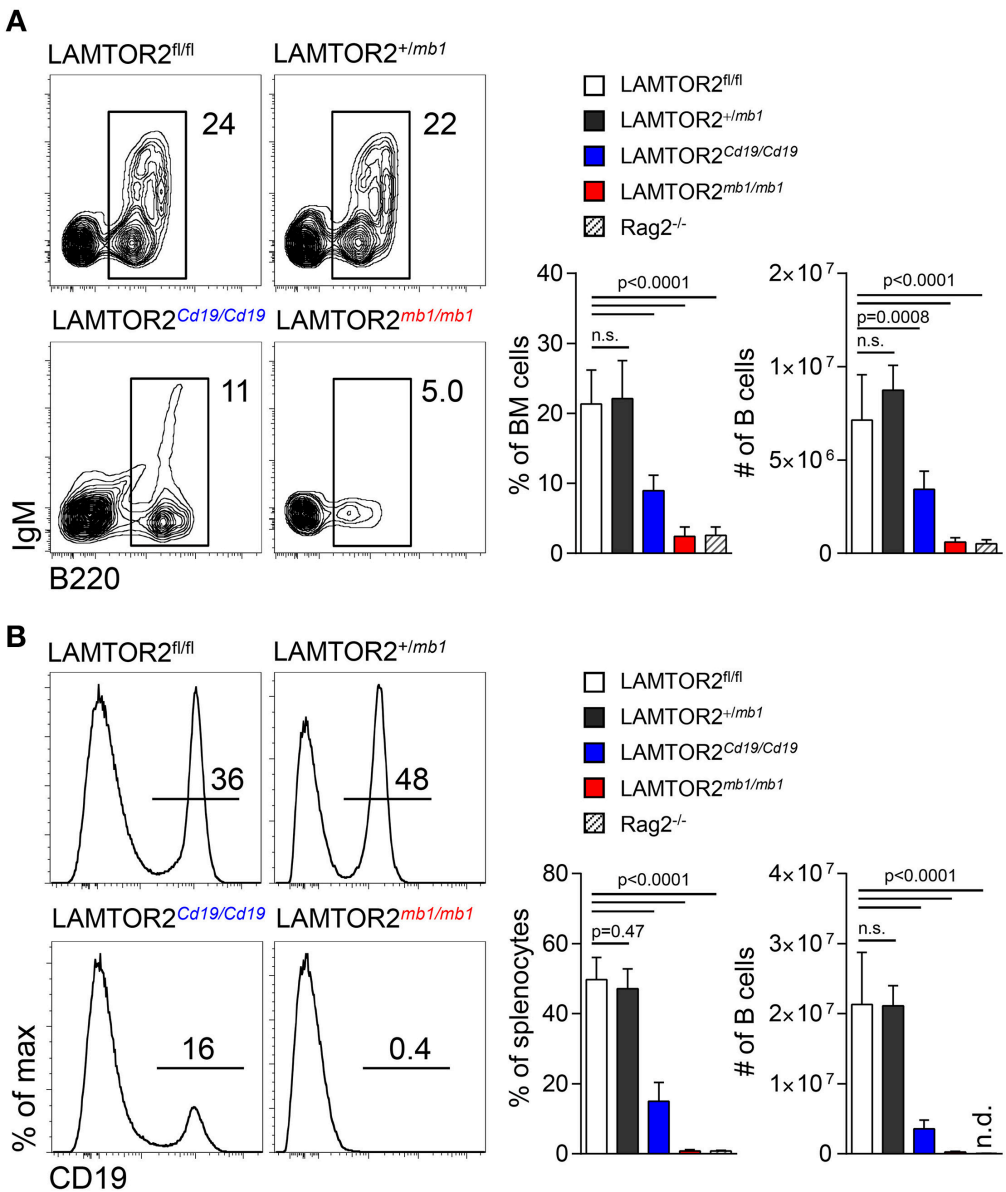
LAMTOR2 is critical for B-cell development. **(A)** FACS analysis of B cells in bone marrow of LAMTOR2^fl/fl^, LAMTOR2^+/mb1^, LAMTOR2^Cd19/*Cd19*^, and LAMTOR2^mb1/*mb1*^ mice. Representative plots of multiple experiments (left panel) are depicted. Percentage within all BM cells and total number of pooled two independent experiments (right panel) are shown (mean + SD, *n* = 6–13, Rag2^−/−^ *n* = 3). **(B)** Analogous to A, flow cytometric analysis of B cells in spleen of LAMTOR2-deficient mice. Representative histograms of multiple experiments are shown (left). Frequency of B cells within all splenocytes and total number of B cells from two independent experiments (right panel, mean + SD, sample numbers: *n* = 6–13, Rag2^−/−^ *n* = 3). Statistical analysis was performed using unpaired *t*-test.

To assess the functional role of LAMTOR2 in B cells we crossed mice carrying floxed alleles of LAMTOR2 with two different Cre deleter strains. CD19-Cre mice are comparatively inefficient deleters in developing B cells but display continuous Cre expression throughout the B lineage starting at the pre-B1 stage (15, 16). In contrast, mb1-Cre mice delete floxed alleles very efficiently also starting essentially at the pre-B1 stage (17). CD19-Cre-mediated deletion of LAMTOR2 (termed LAMTOR2^*Cd*19/*Cd*19^ here) resulted in an ~2-fold reduced frequency and absolute number of B lineage cells in bone marrow (BM) (Figure 1A). In spleen, frequencies and absolute numbers of B cells were further reduced in the absence of LAMTOR2 (Figure 1B). Mice carrying a loss of LAMTOR2 through mb-1-mediated deletion (termed LAMTOR2^*mb*−1/*mb*−1^ here) lacked B lineage cells altogether, showing virtually identical numbers of B220 and CD19 positive cells as Rag2^−/−^ mice, which display a complete block in immunoglobulin (Ig) gene rearrangement (Figures 1A,B). The observed difference between the two Cre deleter strains was not caused by heterozygous modification of the mb-1 locus (encoding the BCR signaling component CD79a), as mb1-Cre-positive mice heterozygous for the floxed LAMTOR2 allele had essentially normal numbers of B cells in BM and spleen (Figures 1A,B).

### LAMTOR2 Is Required for the Pre-B1-to-Pre-B2 Developmental Transition

**Figure 2:**
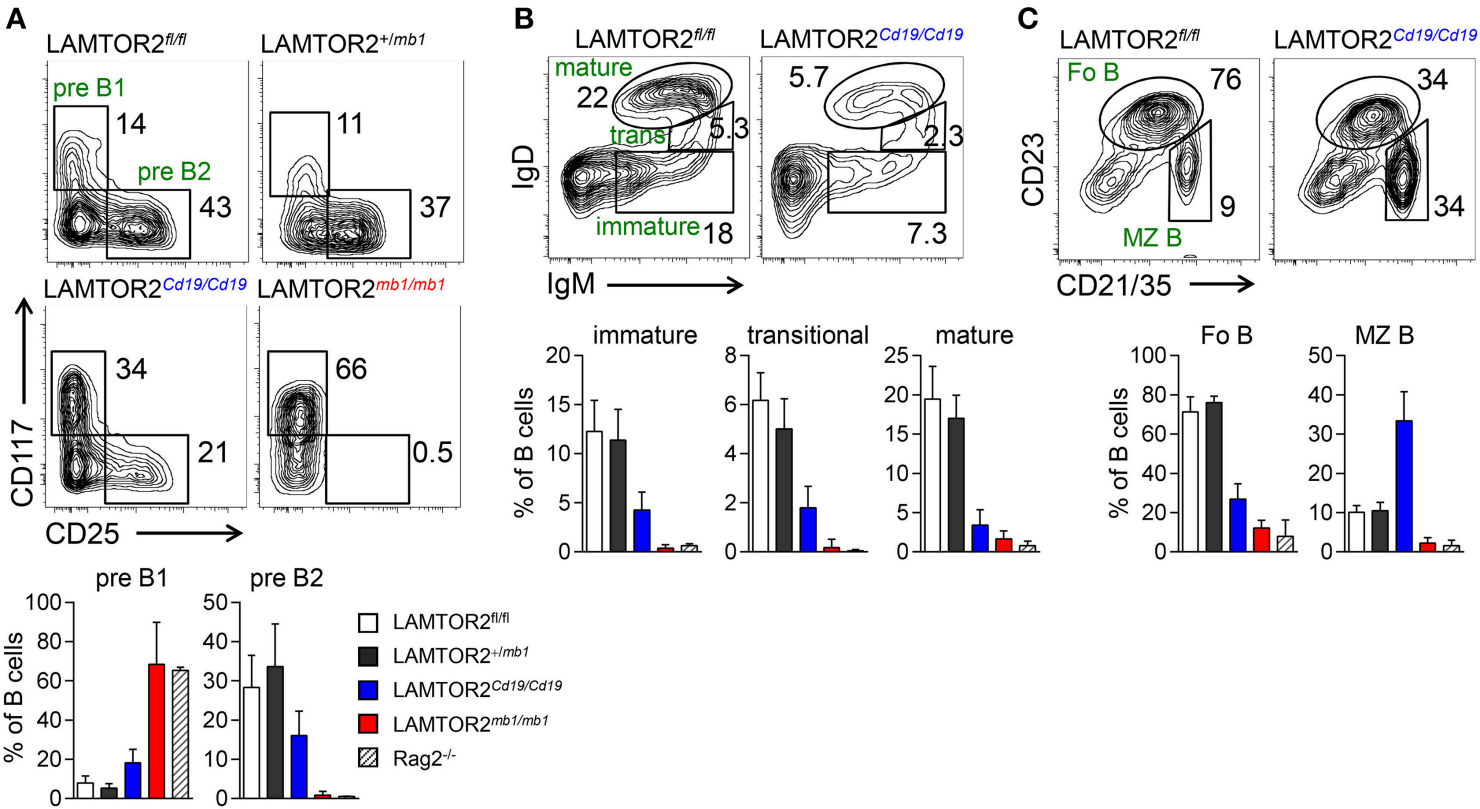
LAMTOR2 is required for the pre-B1-to-pre-B2 developmental transition. **(A)** FACS plots and quantification of pre-B1, pre-B2 cells in BM from LAMTOR2^fl/fl^, LAMTOR2^+/*mb1*^, LAMTOR2^Cd19/*Cd19*^, and LAMTOR2^mb1/*mb1*^ mice (pre-B1: B220^lo^CD19^+^CD117^+^CD25^−^; pre-B2: B220^lo^CD19^+^CD117^−^CD25^+^). **(B)** FACS plots and quantification of immature, transitional, and mature B cells in BM from LAMTOR2^fl/fl^ and LAMTOR2^Cd19/*Cd19*^ mice (immature B: B220^lo^CD19^+^IgM^+^IgD^−^; transitional B: B220^lo−hi^CD19^+^IgM^+^IgD^lo^; mature B: B220^hi^CD19^+^IgM^+^IgD^hi^). **(C)** Analogous to B, flow cytometric analysis and quantification of splenic B cells (follicular B: CD19^+^CD23^hi^CD21^lo/−^; marginal zone B: CD19^+^CD23^lo/−^CD21^hi^). **(A–C)** Representative plots of three independent experiments are shown. Populations a redefined next to the respective gates. Numbers adjacent to gates indicate percentages within all B cells. Frequencies in charts are within all B cells. Pooled data of two independent experiments (mean + SD, *n* = 6–13, Rag2^−/−^ *n* = 3). Statistical analysis was performed using unpaired *t*-test.

The transition between pre-B1 and pre-B2 cells depends on productive Ig heavy chain rearrangement and is characterized by loss of CD117 and induction of expression of CD25. LAMTOR2^*mb*−1/*mb*−1^ mice completely lacked pre-B2 cells (showing similar levels as Rag2-deficient mice) but displayed normal numbers of pre-B1 cells (Figure 2A and Figure S1A). These data indicate that LAMTOR2 was strictly required for the developmental transition between pre-B1 and pre-B2 cells. As a consequence, all later stages of B-cell development were equally absent in LAMTOR2-deficient mice (Figures 2B,C and Figures S1A,B). In order to assess the effect of LAMTOR2 deletion on later stages of B cell development, we also analyzed LAMTOR2^*Cd*19/*Cd*19^ mice, which displayed a partial block at the pre-B1-to-pre-B2 transition, indicated by an increased proportion of pre-B1 cells at the expense of pre-B2 cells (Figure 2A). Accordingly, numbers of pre-B2 cells were lower in BM of LAMTOR2^*Cd*19/*Cd*19^ mice (Figure S1). Frequencies and absolute numbers of immature, transitional, and mature B cells were reduced in these mice at a similar degree as pre-B2 cells when compared to wild-type (WT) controls (Figure 2B and Figure S1). In addition, we noted a small shift toward the T1 subset, when directly comparing T1 and T2 transitional B cells (Figure S1C). Whereas, in spleen numbers of follicular B (Fo B) cells were severely affected by deletion of LAMTOR2, numbers of marginal zone B (MZ B) cells were barely reduced, resulting in increased relative proportions of these cells MZ B cells (Figure 2C and Figure S1). In order to test whether the partial block in B cell development observed in LAMTOR2^*Cd*19/*Cd*19^ mice could be directly attributed to inefficient deletion, residual expression of LAMTOR2 mRNA was assessed using qRT-PCR. Indeed, LAMTOR2 transcripts were detected in all B cell subsets analyzed, indicating a range of deletion efficiency between ~50% in Fo B cells to more than 80% in pre-B2, immature and MZ B cells (Figure S2). These data suggest that different degrees of counter-selection against deletion of LAMTOR2 are in place and that Fo B cells are particularly dependent on LAMTOR2.

### LAMTOR2 Regulates Pre-BCR-Dependent Events

**Figure 3:**
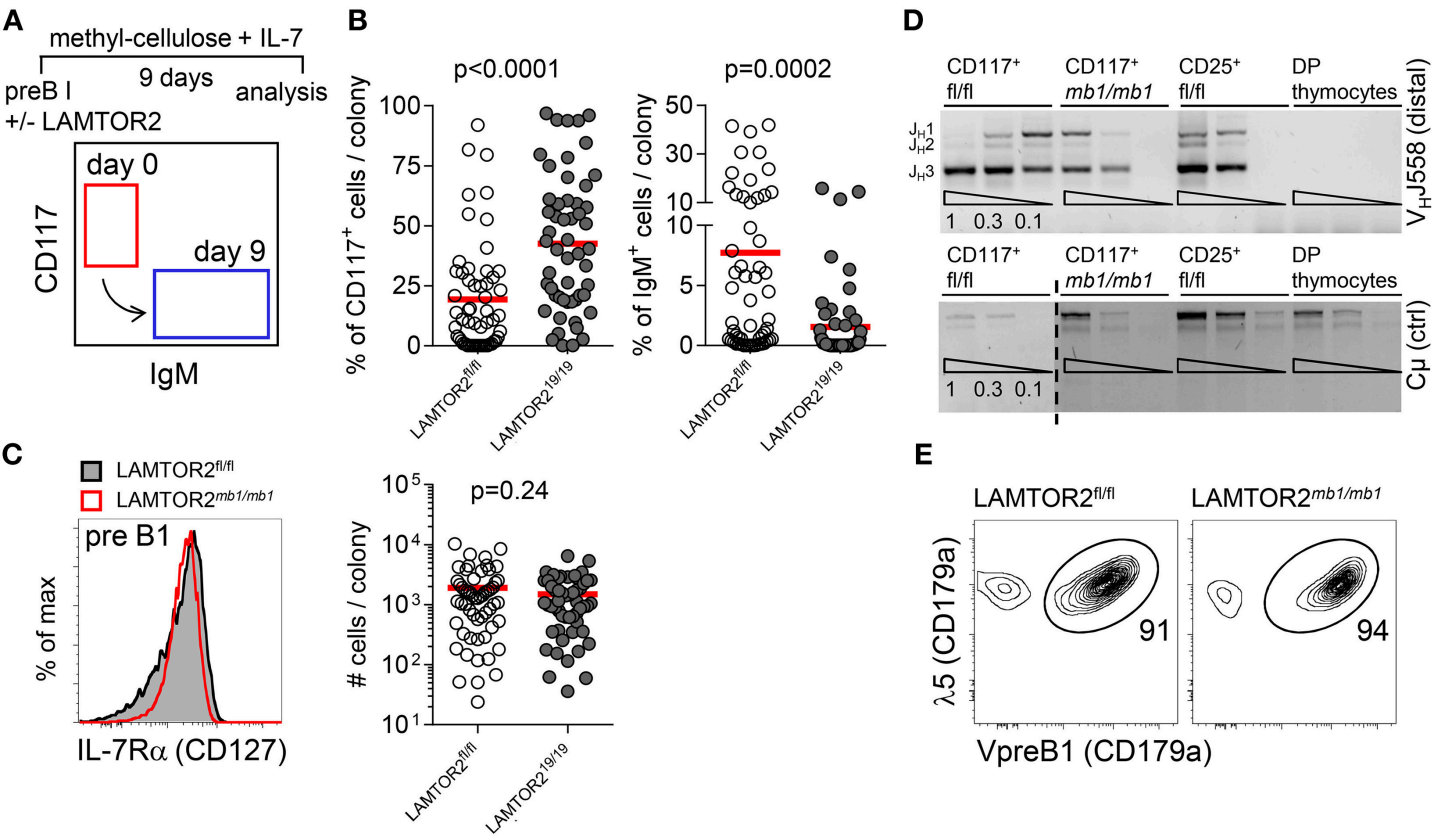
LAMTOR2 is required for differentiation of B cell progenitors but not their expansion. **(A–C)** Pre-B1 cells (B220^+^CD19^+^CD117^+^) were sorted from bone marrow of LAMTOR2^fl/fl^ or LAMTOR2^Cd19/*Cd19*^ mice and cultured on semi-liquid methylcelullose substrate supplemented with murine IL-7. Nine days later cells from individual colonies were collected and analyzed by flow cytometry. **(A)** Experimental scheme and diagram illustrating development of B cell progenitors. **(B)** Percentages of early (B220^+^CD117^+^IgM^−^) and late (B220^+^CD117^−^IgM^+^) B cell progenitors. Each dot represents data from a single colony. **(C)** Flow cytometric analysis of cell surface expression of IL-7Ra (CD127) on pre-B1 cells from bone marrow of LAMTOR2^fl/fl^ or LAMTOR2^*mb*1/*mb*1^ mice (left panel) and analysis of proliferative expansion of B cell progenitors (LAMTOR2^fl/fl^ or LAMTOR2^Cd19/*Cd19*^) cultured on methylcellulose in the presence of IL-7. Individual colonies were retrieved from methylcellulose and quantitated by FACS. The y-axis denotes number of cells per isolated colony. **(D)** PCR analysis of genomic DNA for V_H_J558-to-J_H_3 rearrangements from sorted pre-B1 cells. pre-B2 (B220^+^CD19^+^CD25^+^) from LAMTOR2^fl/fl^ mice cells were used as recombination positive control and thymocytes as negative control. Expression of recombination-independent C serves as loading control. PCRs were carried out on the DNA content of 60,000, 20,000, and 6,700 cells. **(E)** Intracellular staining of surrogate light chain components (VpreB1 and λ5) expressed by pre-B1 cells. **(B,C)** Pooled data of two independent experiments are shown. Each dot represents data from a single colony. **(D)** Results of an individual experiment are shown; FACS plots on **(C,E)** are representative of two independent experiments. Statistical analysis was performed using unpaired *t*-test.

In order to address the molecular mechanism, by which LAMTOR2 regulates B cell development we employed methylcellulose cultures of pre-B1 cells in the presence of IL-7. After 9 days of culture, colonies were assessed for expression of IgM (Figure 3A). In this assay expansion of colonies is dependent on IL-7 and differentiation into IgM^+^ cells depends on signaling through the pre-BCR, thus allowing us to discriminate between the two central signaling pathways that are critical for developmental progression at this stage. Whereas, in control cultures on average 20% of cells had retained expression of CD117, in cultures from LAMTOR2^*Cd*19/*Cd*19^ cells, 40% had retained a pre-B1 phenotype. In turn, control colonies contained on average 8% IgM^+^ cells, whereas LAMTOR2-deficient colonies had generated on average 2% IgM^+^ cells (Figure 3B). Interestingly, the number of cells per colony was not altered in the absence of LAMTOR2 (Figure 3B). In line, expression of IL-7Rα was not affected by loss of LAMTOR2 (Figure 3C). Next, we tested whether LAMTOR2 contributed to *Ig* gene rearrangement. PCR-based analysis revealed no major differences between distal VDJ_H_ rearrangements in control and LAMTOR2^*mb*−1/*mb*−1^ pre-B1 cells (Figure 3D). Expression of the pre-BCR surrogate light chains, λ5, and VpreB1, was also comparable between LAMTOR2-sufficient and LAMTOR2-deficient pre-B1 cells (Figure 3E). Taken together, these data indicate that LAMTOR2 was not required for IL-7 signaling or expression of the pre-BCR. Therefore, by excluding these mechanisms it is most likely that LAMTOR2 controls pre-BCR signaling and that these effects at least partially account for the developmental block in the absence of LAMTOR2.

### LAMTOR2 Orchestrates BCR Downstream Signaling Pathways

**Figure 4:**
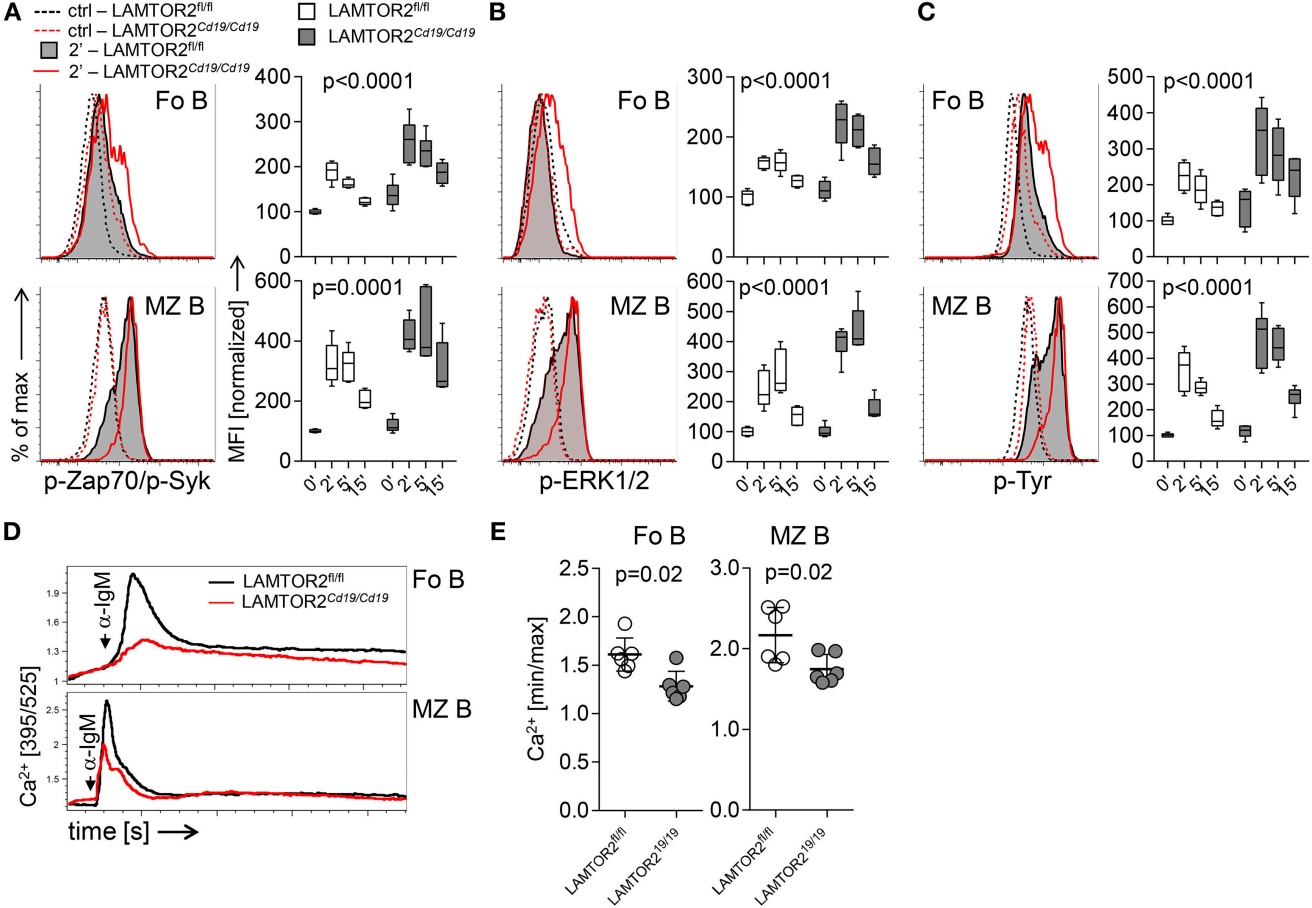
Aberrant BCR-associated signaling in LAMTOR2-deficient B cells. Flow cytometric analysis of **(A)** pZap70 (pY319)/pSyk (pY352) **(B)** pERK1/2 (pT202/pY352), and **(C)** total pY in splenic B cells isolated from LAMTOR2^fl/fl^ or LAMTOR2^Cd19/*Cd19*^ mice. Starved splenocytes were left untreated (ctrl) or stimulated with anti-IgM F(ab′)_2_ for 2, 5, and 15 min. Representative histograms of three independent experiments are shown, graphs show summarized data of two independent experiments, *n* = 6 for each genotype, whiskers indicate min. to max. range of data, horizontal bars show mean value. Median fluorescence values (MFI) were normalized to ctrl (set as 100%). Statistical analysis was performed using two-way ANOVA (*p*-values for effect of genotype). **(D)** Total splenocytes were stimulated with anti-IgM F(ab′)_2_ and Ca^2+^-flux was recorded flow cytometrically over time. Plots for electronically gated Fo B cells and MZ B cells are representative for 6 (ctrl) or 7 (ko) mice from two individual experiments. “anti-IgM” indicates time point of stimulation. **(E)** Bar graph shows analysis of peak Ca^2+^ flux over background from two independent experiments, each dot represents one mouse. Statistical analysis was performed using unpaired *t*-test.

In order to test whether LAMTOR2 modulated signaling through the BCR we took advantage of the partial B-lineage developmental block in LAMTOR2^*Cd*19/*Cd*19^ mice. First, we analyzed phosphorylation of Erk (pErk), Syk (pSyk), and tyrosines in general (pY) after BCR triggering in both Fo B and MZ B cells. Unexpectedly, in the absence of LAMTOR2 we observed higher levels of pSyk (Figure 4A), pErk (Figure 4B), and pY (Figure 4C) after BCR triggering of both Fo B and MZ B cells when compared to controls. Co-treatment with H_2_O_2_, to inhibit phospho-tyrosine phosphatase activity, showed similar results, indicating that LAMTOR2 directly curtails certain phosphorylation events rather than promoting dephosphorylation (Figure S3). Next, we analyzed Ca^2+^-flux after BCR triggering in both B cell populations. In contrast to the increase in phosphorylation, levels of Ca^2+^-flux were reduced after BCR triggering in LAMTOR2-deficient Fo B and MZ B cells when compared to controls (Figures 4D,E). Note, that these effects are based on mixed populations, in which not all cells, in particular within the Fo B cell population, have lost expression of LAMTOR2. Therefore, the effects of deletion of LAMTOR2 are most likely more pronounced than indicated here. In summary, these data indicate that loss of LAMTOR2 results in a disbalance of BCR-triggered signaling pathways rather than overall weakening or strengthening of the BCR signal. As the pre-BCR shares many features of the signaling machinery with the BCR, these findings also further support the notion that the early developmental block in the absence of LAMTOR2 is, in part, due to aberrant pre-BCR signaling.

### Loss of LAMTOR2 in Peripheral B Cells Results in Impaired BCR-Mediated Expansion

**Figure 5:**
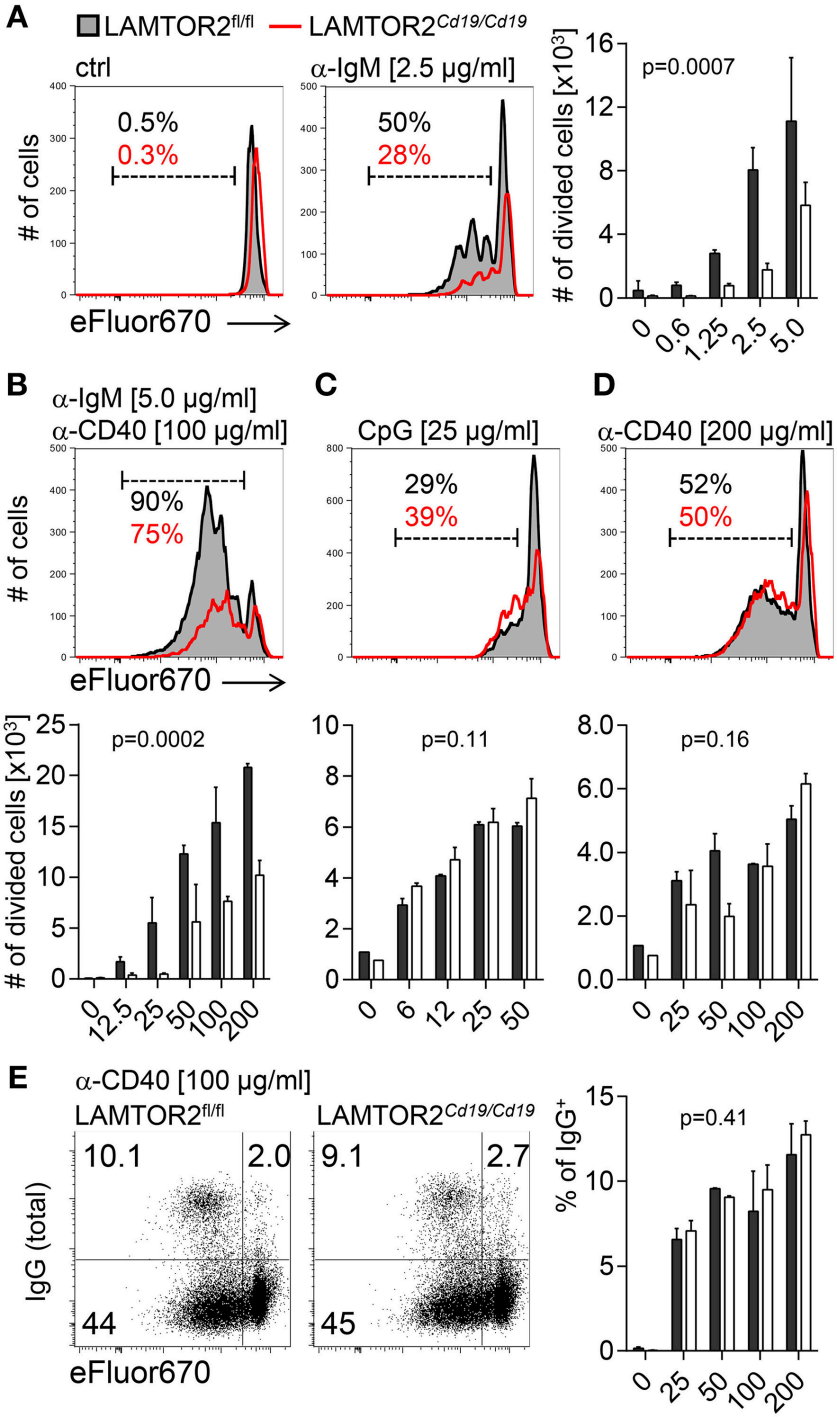
Aberrant expansion of LAMTOR2-deficient B cells in response to B-cell receptor stimulation. Splenic follicular B cells isolated from LAMTOR2^fl/fl^ or LAMTOR2^Cd19/*Cd19*^ mice were stained with cell proliferation dye and stimulated with **(A)** polyclonal anti-IgM F(ab′)_2_ fragments; **(B)** anti-IgM F(ab′)_2_, and anti-CD40 Ab; **(C)** CpG or **(D)** anti-CD40 Ab. B cell proliferative response was analyzed by flow cytometry 2 (CpG) or 3 (all other conditions) days after stimulation. Gates in histograms and adjacent numbers indicate the frequency of divided cells. **(E)** Frequency of IgG^+^ class switched B cells was measured within divided cells. FACS plots and adjacent chart are representative of two (CpG) or three independent experiments. Bars represent SD. Statistical analysis was performed using two-way ANOVA (*p*-values for effect of genotype).

Next, we tested functional consequences of disbalanced BCR signaling upon loss of LAMTOR2. *In vitro* expansion was analyzed 3 days after triggering with increasing concentrations of anti-IgM antibodies. LAMTOR2-deficient B cells expanded less when compared to controls at every indicated concentration of stimulus (Figure 5A). The defect in BCR-dependent expansion could not be compensated by increasing levels of anti-CD40-mediated co-stimulation (Figure 5B). Accordingly, expansion induced by CpG or by CD40 triggering alone were unaffected by loss of LAMTOR2 in B cells (Figures 5C,D). Furthermore, LAMTOR2-deficiency did not impair CD40-mediated Ig class-switch (Figure 5E). Thus, we conclude that a disbalance in BCR downstream signaling results in impaired BCR-mediated expansion, which could not be rescued by triggering of additional proliferative signals.

### LAMTOR2 Regulates Internalization and Intracellular Trafficking of the BCR

**Figure 6:**
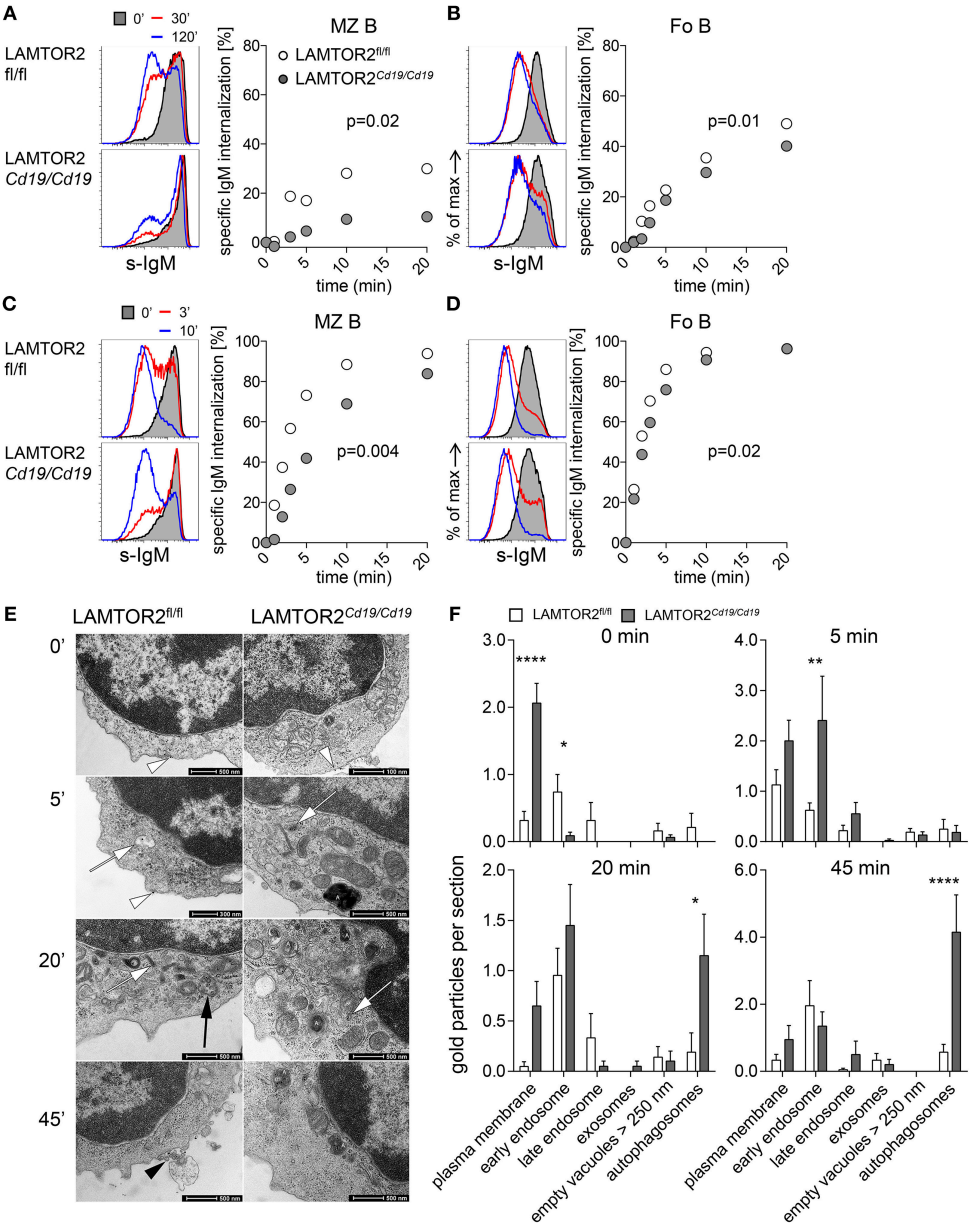
Altered internalization and degradation of BCR in LAMTOR2-deficient B cells. FACS analysis of BCR internalization in splenic B cells. Passive BCR internalization in **(A)** MZ B, **(B)** Fo B cells or ligand-induced BCR internalization in **(C)** MZ B or **(D)** Fo B cells. Purified splenic B cells were labeled in an ice-cold environment with either monovalent **(A,B)** or bivalent **(C,D)** biotin-labeled anti-IgM Fab or F(ab′)_2_ fragments, respectively, and then BCR internalization was assessed over time at 37°C. Plots for electronically gated cells are representative for two independent experiments. Charts summarize the first 20 min of one representative experiment out of two. **(E)** Transmission electron microscopy (TEM) analysis of ligand-induced BCR internalization and degradation in B cells of LAMTOR2^fl/fl^ or LAMTOR2^Cd19/*Cd19*^ mice. Purified B cells were stimulated with colloidal-gold labeled anti-IgM F(ab′)_2_ and analyzed at indicated time points. White arrowheads demonstrate gold particles at the cell membrane, white arrows mark early endosomes with gold-marked IgM receptors, the black arrow shows the late endosome with gold at the internal vesicles, the black arrowhead marks exosomes with attached gold particles. Autophagosomes are labeled with an “A.” **(F)** Quantification of TEM data. The quantitative evaluation has been done only at cells cut through the cell center to get access directly to all cell organelles. Nineteen to thirty-eight sections per time point and genotype were analyzed. Statistical significance was assessed with 2-way ANOVA (effect for genotype is shown) and Sidak's multiple comparison test (^*^p < 0.05; ^**^p < 0.01; ^****^p < 0.001).

Given the role of LAMTOR2 as endosomal adapter protein, we hypothesized that disbalanced BCR signaling in the absence of LAMTOR2 might be due to alterations in BCR trafficking after stimulation. To test the hypothesis we first analyzed stimulation-dependent internalization of the BCR *in vitro*. To this end, surface BCR was labeled with anti-IgM antibodies in the cold. To assess passive internalization of the BCR cells were labeled with monovalent anti-IgM fragments in the cold and cultured at 37°C without additional stimulation. Both MZ B (Figure 6A and Figure S4A) and Fo B cells (Figure 6B and Figure S4A) from LAMTOR2^*Cd*19/*Cd*19^ mice retained more BCR on their surface over a period of 120 min when compared to WT controls. Stimulation-dependent BCR internalization was assessed in a similar assay replacing monovalent by bivalent anti-IgM fragment in order to induce BCR crosslinking. Similarly, MZ B (Figure 6C and Figure S4B) and Fo B cells (Figure 6D and Figure S4B) from LAMTOR2^*Cd*19/*Cd*19^ showed delayed internalization of the BCR when compared to WT controls.

Next, we employed transmission electron microscopy to assess intracellular trafficking of the BCR upon stimulation using immunogold labeling. Consistent with our finding that passive internalization was impaired in the absence of LAMTOR2 (Figures 6A,B), BCRs were detected in multiple subcellular compartments prior to stimulation in controls, whereas in B cells from LAMTOR2^*Cd*19/*Cd*19^ mice BCRs were predominantly detected on the cell surface (Figures 6E,F). Within 5 min of stimulation significantly more BCRs were detectable in early endosomes from LAMTOR2^*Cd*19/*Cd*19^ B cells when compared to controls, followed by accumulation in autophagosomes at 20 and 45 min after stimulation. Of note, such autophagosomes were almost absent from wild-type B cells and the frequency of B cells with a high abundance of autophagosomes in samples from LAMTOR2^*Cd*19/*Cd*19^ mice correlated well with the penetrance of LAMTOR2 deletion as detected by qRT-PCR. Given that in the absence of LAMTOR2, BCR trafficking was already altered at steady state, these data suggest that loss of LAMTOR2 perturbed intracellular localization of the BCR, which, in turn, resulted in aberrant BCR signaling.

## Discussion

Here we demonstrated that the endosomal adaptor protein LAMTOR2 is essential for B cell development at the pre-BCR checkpoint. Loss of LAMTOR2 resulted in aberrant BCR signaling, a defect in BCR internalization and aberrant intracellular trafficking. These data highlight the critical importance of spatial organization of signaling modules in biological processes.

It has been previously described in multiple studies that inhibition of BCR internalization by various means results in aberrant signaling. Blocking of endocytosis resulted in increased tyrosine phosphorylation as well as increased and sustained activation of MAP kinase pathways (8, 18). In contrast, activation of the PI3-K/Akt pathway was inhibited (8). Mutation of the ITAMs in Igβ also leads to dysregulation of BCR internalization, although in this case steady-state turnover is more strongly affected than signal-induced endocytosis (19). However, despite elevated Ca^2+^ responses, sustained Erk activation and overall tyrosine hyperphosphorylation upon mutation of Igβ ITAMs, hypophosphorylation of Syk and increased activation of the PI3-K pathway were observed. Loss of LAMTOR2 analyzed here resulted in general hyperphosphorylation of Syk, Erk, and overall tyrosines but a limited Ca^2+^ response. Together, these studies imply that BCR signaling is tightly regulated through receptor internalization and compartmentalization of signaling. Opposing outcomes in different signaling modules dependent on variations in experimental setup highlight the intricate balance maintained by compartmentalization of signaling. The dual function of LAMTOR2 as mediator of endosomal trafficking as well as retention factor for the Erk module at late endosomes at present precludes a definitive conclusion as to whether spatial dislocation of Erk or aberrant BCR trafficking or both are key for aberrant signaling in the absence of LAMTOR2. Differences in passive BCR internalization in the absence of LAMTOR2 may suggest that aberrant BCR trafficking determines signaling outcomes. In addition, it has also been reported that conditional ablation results in defective homeostasis of dendritic cells due to accumulation of Flt3 on the cell surface followed by downstream activation of Akt/mTOR signaling (20). This study indicated that aberrant endocytosis can directly result in uncoupling of signaling cascades. Alterations in endosomal trafficking in the absence of LAMTOR2 can be ascribed to destabilization of the complete LAMTOR/Ragulator complex. This complex has been reported to integrate mTOR signals which might control the composition of intracellular compartments based on metabolic needs (21). In addition, it has recently been reported that the LAMTOR/Ragulator complex is critically involved in late endosomal positioning (22). Furthermore, our observation is also consistent with a previous study directly analyzing the consequences of inhibition of endocytosis on BCR signaling (8). However, we cannot exclude that differences in tonic signaling prior to B cell isolation contribute to alterations in passive BCR internalization.

Despite the defect in BCR internalization, mutation of the ITAMs in Igβ did not result in major developmental defects (19). In contrast, we observed a complete developmental block at the pre-BCR checkpoint suggesting that the pre-BCR is particularly sensitive to aberrant compartmentalization of signaling modules. This hypothesis is consistent with the observation that only a low percentage of pre-BCR is located at the cell surface at any given time and that turnover of the pre-BCR is very high with rates of internalization of 40% within 5 min (23). However, because of low levels of surface expression, alterations of pre-BCR turnover remain difficult to quantitate in primary cells. Compared to the virtually complete block in B-cell development upon deletion of LAMTOR2, defects in BCR signaling and internalization were comparatively mild. We cannot exclude that additional signaling pathways active at the pre-BCR checkpoint were also affected by disruption of the LAMTOR complex. Signaling through the IL-7R is the second major pathway at the pre-BCR checkpoint. However, neither clonal analysis *in vitro* nor surface expression of IL-7R suggest that this pathway was critically affected by loss of LAMTOR2. In peripheral B cells, loss of LAMTOR2 limited anti-IgM-mediated, but not anti-CD40-mediated proliferation, further indicating that BCR, and pre-BCR are the major pathways controlled by the LAMTOR complex in the B lineage. Of note, most functional experiments were performed in B cells from mice with Cd19-Cre-mediated deletion of LAMTOR2. In these mice the B-lineage developmental defect was considerably milder than in mice with mb-1-Cre-mediated deletion of LAMTOR2. This difference was due to inefficient deletion, with substantial counter-selection becoming apparent in Fo B cells.

In the periphery, Fo B cells were more strongly affected by loss of LAMTOR2 than MZ B cells. Using a Nur77-GFP reporter to monitor BCR signal strength, it was shown that MZ B cells respond differently than Fo B cells to tonic signals modulated by an allelic series of CD45 expression (24). Although it remains an open question how the complex alterations in BCR downstream signaling upon loss of LAMTOR2 translate into tuning of BCR signal strength, these differences may explain why MZ B cells were less affected by LAMTOR2-deficiency than Fo B cells.

In human patients with homozygous mutations in LAMTOR2, no complete block in B cell development but rather a defect in class-switched B cells associated with hypogammaglobulinemia was observed (14). In contrast to the knockout mice, the human mutation affected the 3′-UTR and allowed for residual protein expression (14). Nevertheless, defects in Ig class-switch recombination as well as B-cell memory formation support the idea that p14 expression controls BCR signaling in patients as well.

Thus, LAMTOR2/p14 is essential in controlling BCR trafficking and highlights a non-redundant role for the LAMTOR-complex in securing physiological differentiation of B cells.

## Materials and Methods

### Mice

All animal experiments were conducted in accordance with local and institutional regulations. C57BL/6J and Rag2^−/−^ mice were purchased from Charles River or bred at the animal facilities of Hannover Medical School and LMU Munich. LAMTOR2^fl/fl^ mice (10) were crossed with CD19cre^ki/wt^ (15) (termed LAMTOR2^Cd19/Cd19^ here) and mb-1-Cre (17) (LAMTOR2^mb−1/mb−1^) and maintained at Hannover Medical School and LMU Munich. Animals were maintained under specific-pathogen-free conditions.

### Flow Cytometry

Flow cytometry and cell sorting were performed on LSRII and FACSAriaIIu (BD) cytometers, respectively. Monoclonal antibodies specific for IgD (clone 11-26c), IgM (II/41), CD23 (B3B4), CD21/35 (7E9), CD179a (R3), CD179b, p-Zap70/p-Syk (p-Y319/p-Y352; 17A/P-ZAP70), p-ERK1/2 (pT202/pY204; 20A), p-Tyr (pY20), CD25 (PC61), Gr-1 (RB6-8C5), erythroid cell marker (Ter-119), CD19 (1D3), CD11b (M1/70), NK1.1 (PK136), B220 (RA3-6B2), CD117 (ACK2) were used purified or as various fluorescent or biotin conjugates. Antibodies were purified from hybridoma supernatants or were purchased from eBioscience, BD Biosciences, BioLegend, or Miltenyi Biotech. Flow cytometry data were analyzed in FlowJo (9.3, TreeStar).

Bone marrow B cell stages were defined as follows: pre-BI (B220^+^CD19^+^CD117^+^CD25^−^), pre-B2 (B220^+^CD19^+^CD25^+^CD117^−^), immature B (CD19^+^IgM^lo^IgD^−^), transitional (CD19^+^IgM^hi^IgD^+/−^), mature (CD19^+^IgM^int/−^IgD^+^). Splenic B cell stages were defined as: Fo B cells (CD19^+^CD23^+^CD21/35^−^), and MZ B cells (CD19^+^CD23^−^CD21/35^+^).

### *Ex vivo* Stimulation of B Cells

Single-cell suspension of splenocytes from LAMTOR2^fl/fl^ and LAMTOR2^Cd19/Cd19^ mice were incubated for 30–45 min in FCS free RPMI at room temperature (RT) at 10^7^/mL. Next, splenocytes were incubated with or without stimulation as indicated in the figure legends. Cells were stimulated with anti-IgM F(ab′)_2_ at 10 μg/mL. Reactions were stopped by adding cell suspensions to formaldehyde at 1.5% final concentration. After 15 min of incubation at RT fixed cells were washed and permeabilized with ice-cold methanol for 30 min. Cells were then washed twice to remove residual methanol and stained for B220, CD23, and IgM as well as with phospho-specific antibodies for 30′ at RT. The protocol was adapted from (25).

### Purification of B-Cell Progenitors

Lin^−^ cells were isolated from total BM by staining cell suspensions with a lineage-specific antibody cocktail including TCRβ, IgM, CD11b, CD11c, Gr-1, Ter-119, and NK1.1 (all e-Biosciences or BioLegend) followed by incubation with sheep anti-rat IgG conjugated to magnetic beads (Dynal, Invitrogen) and immunomagnetic depletion of mature lineages. Pre-purified cells were subjected to FACS sorting.

### Measurement of Intracellular Ca2^+^ Flux in Splenic B Cells

Single-cell suspensions of splenocytes from LAMTOR2^fl/fl^ and LAMTOR2^Cd19/Cd19^ mice were incubated for 1 h in Ca^2+^ and Mg^2+^ -free Dulbecco's serum-free medium (Invitrogen) at room temperature at 10^7^ cells/mL. Splenocytes were then loaded with Fluo-4 (3 μM) and FuraRed (6 μM) for 45 min at 37°C. Further, cells were washed and stained for CD21 and CD23/35 for 15 min at 4°C. Subsequently, cells were rested for 30 min at 37°C. After establishing of a baseline for 30 s, B cells were stimulated with anti-IgM F(ab′)_2_, and data acquisition was continued for 4 additional minutes. To ensure cell viability, 30 s before the end of acquisition 2 μg/mL ionomycin (Sigma) was added as positive Ca^2+^-flux control.

### B Cell Proliferation Assay

Primary splenic B cells from LAMTOR2^fl/fl^ and LAMTOR2^Cd19/Cd19^ mice were sorted and cultured at 37°C, 5% CO_2_. B cells were kept in complete alpha-MEM supplemented with 10% FCS and IL-4 (10 ng/ml) and stimulated with cross-linking 20 μg/mL F(ab′)2 fragment goat anti-mouse IgM (Jackson Immuno Research), and/or anti-CD40 (clone FGK4T) or fully thiolated CpG 2006 type B (24 mer 5′-TCGTCGTTTTGTCGTTTTGTCGTT-3′, TIB MOLBIO). Proliferation was assessed by staining of cells with Cell Proliferation Dye eFluor 670 according to manufacturer's instructions (eBioscience).

### Methylcellulose Assay

Clonal expansion of pre-B1 cells was assessed in methylcellulose containing IL-7 (MethoCult M3630, Stemcell Technologies) according to the manufacturer's instructions with a starting cell number of 5,000–20,000 sorted cells. Cellularity and cell differentiation was assessed by picking single colonies and flow cytometric cell counting and analysis after 9 days. Cells expressing CD117 were considered as non-differentiated early progenitors, IgM positive cells were taken as differentiated cells.

### BCR Internalization

BCR internalization was assessed on purified splenic B cells. Passive internalization was assessed on MZ B or Fo B cells labeled in an ice cold environment with monovalent anti-IgM–biotin Fab fragments (Jackson Immuno Research). To assess active (ligand induced) internalization of BCR cells were labeled with anti-IgM F(ab′)_2_-biotin particles. After labeling cells were kept at 37°C for the indicated time, followed by fixation and detection of remaining BCR molecules with streptavidin-APC (eBioscience).

### Gene Expression and Rearrangement PCR

V_H_J558 to DJ_H_3 rearrangement was assessed on genomic DNA. The genes belonging to the V_H_J558 family are located at the most 5′ end of the V_H_ locus and belong to the most frequently used V_H_ families (26–28).Genomic DNA was isolated using QIAamp micro kit (Qiagen) and the PCR was carried out on 60,000; 20,000; and 6,700 cells using 1 nM of primers as previously described (29, 30) in 1x PCR reaction buffer (B9004S, Thermopol) for 40 cycles (annealing 59°C).

### Transmission Electron Microscopy Analysis

Isolated B cells pooled of LAMTOR2^fl/fl^ and LAMTOR2^Cd19/Cd19^ mice were incubated at 4°C firstly with biotin SP-conjugated affiniPure F(ab′)_2_-fragment goat anti-mouse IgM followed by staining with Streptavidin-20 nm gold conjugates. The reaction was stopped immediately (0 min) and after warming up at 37°C for 5 min, 20 min as well as 45 min by fixation in 2.5% glutaraldehyde in sodium cacodylate buffer and postfixation in 2% osmium tetroxide in the same buffer. After dehydration in graded ethanol pellets were embedded in Epon. Thin sections stained with 2% uranyl acetate and lead citrate were analyzed with the Tecnai G2 200 kV. Random sections of the cell center have been selected. Because of different penetrance of the CD19-cre KO cells only cells containing autophagosomes have been analyzed (resting B cells sufficient for LAMTOR2 contain a very low number of autophagosomes) in three independent experiments. Notably, frequencies of autophagosome-containing cells corresponded almost perfectly to the penetrance of LAMTOR2 deletion assessed by qRT-PCR. The position of the gold particles at the plasma membrane, inside the early endosomes, late endosomes, at the exosomes attached at the plasma membrane, inside empty vacuoles with a diameter larger than 250 nm as well-autophagosomes was counted separated and listed as number per cell. To avoid bias during the analysis all sample labels were temporally removed and organelles containing gold particles were identified by a person not involved in the study.

### Statistics

Statistical significance of differences between two groups was analyzed using paired or non-paired *t*-tests where applicable. For comparison of multiple groups statistical significance was determined using two-way ANOVA and *p*-values for effect of genotype are shown. ANOVA analysis was followed by Sidak's multiple comparison test to assess differences between groups. To avoid bias during TEM analysis samples were quantified in blind way by a person not involved in the study.

## Author Contributions

MŁ, DK, NZ, CK, and AK designed the research. MŁ, DK, NZ, GB, JD, and SG performed the research. MŁ, DK, NZ, GB, CK, and AK analyzed the data. EH, MR, and LH provided vital reagents. MŁ, NZ, and AK wrote the manuscript.

### Conflict of Interest Statement

The authors declare that the research was conducted in the absence of any commercial or financial relationships that could be construed as a potential conflict of interest.
