# Supplementary material for: LAMTOR2 (p14) Controls B Cell Differentiation by Orchestrating Endosomal BCR Trafficking
Source: Front Immunol. 2019 Mar 18;10:497. doi: 10.3389/fimmu.2019.00497 (PMC6431647; doi:10.3389/fimmu.2019.00497)
Supplement: Supplementary file 1 [file Data_Sheet_1.PDF]

Supplementary Material

LAMTOR2 (p14) controls B cell differentiation by orchestrating endosomal BCR trafficking

Marcin Łyszkiewicz[1,2,*,†], Daniel Kotlarz [2,*], Natalia Ziętara[1,2,*,†], Gudrun Brandes[3], Jana Diestelhorst[2], Silke Glage[4], Elias Hobeika[5], Michael Reth[6], Lukas A. Huber[7], Andreas Krueger[1,8,#], Christoph Klein[2,#]

Correspondence: andreas.krueger@kgu.de (A.K.); Christoph.Klein@med.uni-muenchen.de (C.K.)

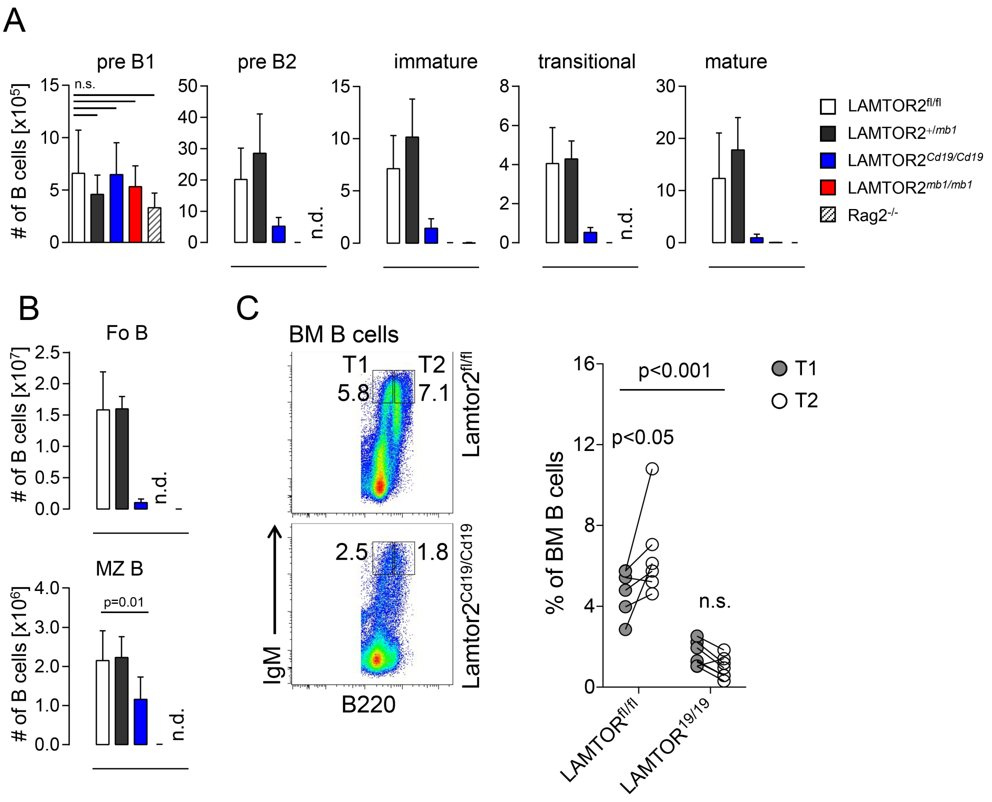

Figure S1 (related to Figure 2). Absolute numbers of B cells in spleen and BM of mice sufficient or deficient for LAMTOR2. (A) Total number of B cell progenitors in BM and (B) main B cell populations in spleen. Pooled data of one or two independent experiments, n = 5-11, $Rag2^{-/-}$ n = 3; n.d. – not detectable. (C) Representative pseudo-colour plots (left) and quantification (right) of transitional 1 (T1) and T2 B cells in BM. Statistical significance was assessed with 2-way ANOVA (effect for genotype is shown) followed by Bonferroni post-test. Pooled data of two independent experiments is shown. Each pair of dots represents data from one mouse.

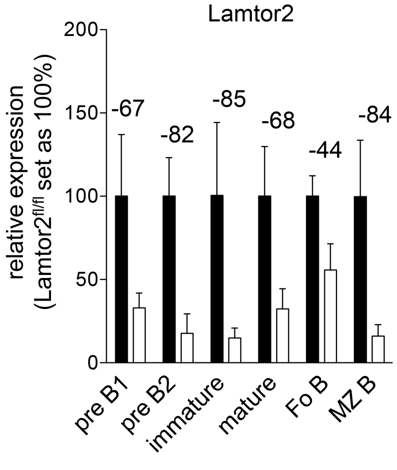

Figure S2. qRT-PCR analysis of CD19-cre mediated deletion of LAMTOR2. Purified populations of BM and splenic B cells of LAMTOR2$^{fl/fl}$ (black bars) and LAMTOR2$^{Cd19/Cd19}$ mice (open bars) were tested for expression of LAMTOR2 mRNA. Average expression in wild-type cells was set as 100%. Numbers above the bars indicate difference in expression between sufficient and deficient cells. Data of one experiment with n=3 for both genotypes is shown.

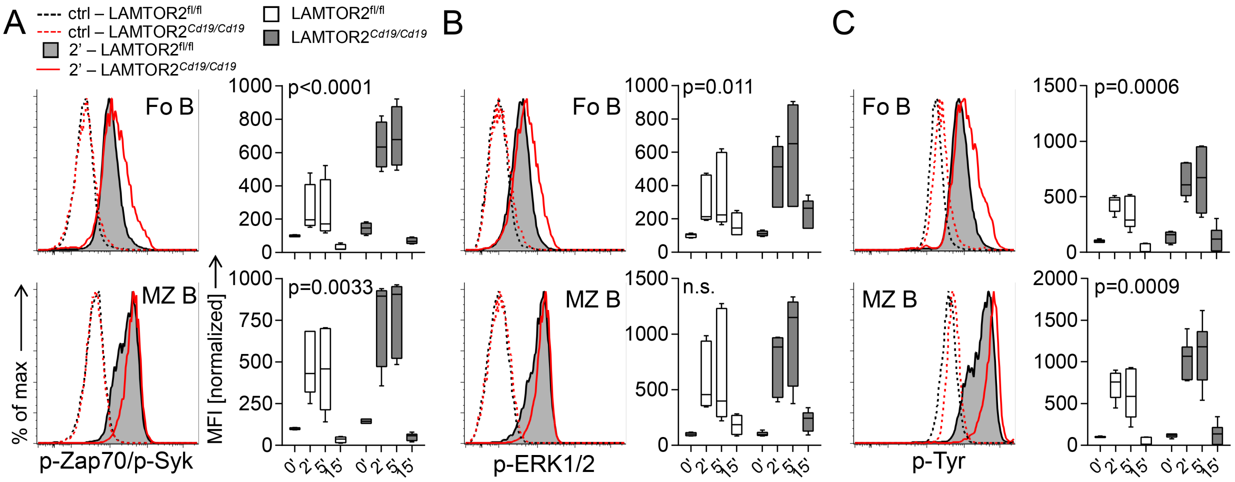

Figure S3 (related to Figure 4). Hyper phosphorylation of BCR-associated kinases in LAMTOR2-deficient B cells. Flow cytometric analysis of (A) pZap70 (pY319)/pSyk (pY352), (B) pERK1/2 (pT202/pY352), and (C) total pY in splenic B cells isolated from LAMTOR2$^{fl/fl}$ or LAMTOR2$^{Cd19/Cd19}$ mice. Starved splenocytes were left untreated (ctrl) or stimulated with anti-IgM F(ab')$_2$ and H_2O_2 for 2, 5 and 15 minutes. Representative histograms of three independent experiments are shown, graphs show summarized data of two independent experiments, n = 6 for each genotype, whiskers indicate min. to max. range of data, horizontal bars show mean value. Median fluorescence values (MFI) were normalized to ctrl (set as 100%). Statistical analysis was performed using two-way ANOVA (p-values for effect of genotype).

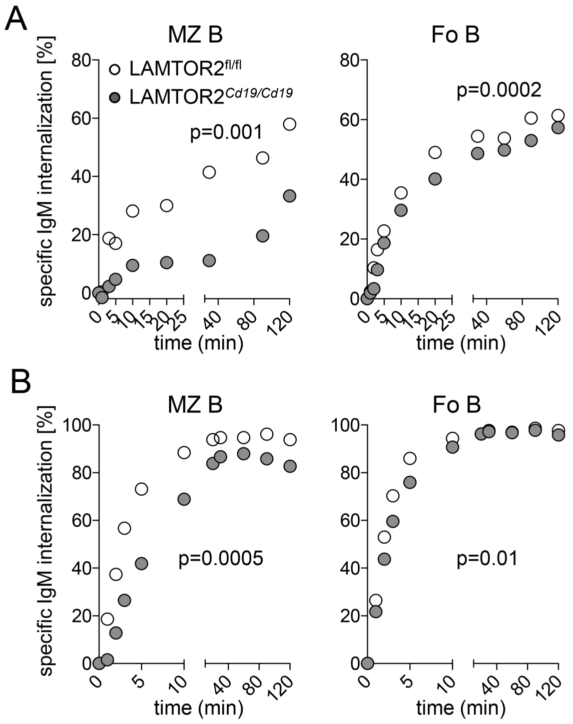

Figure S4 (related to Figure 6). Altered internalization of BCR in LAMTOR2-deficient B cells. Full-time data of the assays presented in Figure 6. (A) Passive BCR internalization in MZ B and Fo B cells or (B) ligand-induced BCR internalization in MZ B or Fo B cells. Purified splenic B cells were labelled in an ice-cold environment with either monovalent (A) or bivalent (B) biotin-labeled anti-IgM Fab or $F(ab')_2$ fragments, respectively, and then BCR internalization was assessed over time at 37°C. Plots for electronically gated cells are representative for two independent experiments. Charts summarize data of one representative experiment out of two.
